# A randomized controlled trial for evaluating the impact of integrating a computerized clinical decision support system and a socially assistive humanoid robot into grand rounds during pre/post-operative care

**DOI:** 10.1177/20552076221129068

**Published:** 2022-09-25

**Authors:** Izidor Mlakar, Urška Smrke, Vojko Flis, Andrej Bergauer, Nina Kobilica, Tadej Kampič, Samo Horvat, Damjan Vidovič, Bojan Musil, Nejc Plohl

**Affiliations:** 1 119108 Faculty of Electrical Engineering and Computer Science, University of Maribor, Maribor, Slovenia; 2 112806 University Clinical Centre Maribor, Maribor, Slovenia; 3 172649 Faculty of Arts, Department of Psychology, University of Maribor, Maribor, Slovenia

**Keywords:** Clinical decision support system, digital health intervention, socially assistive humanoid robots, grand rounds, healthcare quality, quality of life

## Abstract

Although clinical decision support systems (CDSSs) are increasingly emphasized as one of the possible levers for improving care, they are still not widely used due to different barriers, such as doubts about systems’ performance, their complexity and poor design, practitioners’ lack of time to use them, poor computer skills, reluctance to use them in front of patients, and deficient integration into existing workflows. While several studies on CDSS exist, there is a need for additional high-quality studies using large samples and examining the differences between outcomes following a decision based on CDSS support and those following decisions without this kind of information. Even less is known about the effectiveness of a CDSS that is delivered during a grand round routine and with the help of socially assistive humanoid robots (SAHRs). In this study, 200 patients will be randomized into a Control Group (i.e. standard care) and an Intervention Group (i.e. standard care and novel CDSS delivered via a SAHR). Health care quality and Quality of Life measures will be compared between the two groups. Additionally, approximately 22 clinicians, who are also active researchers at the University Clinical Center Maribor, will evaluate the acceptability and clinical usability of the system. The results of the proposed study will provide high-quality evidence on the effectiveness of CDSS systems and SAHR in the grand round routine.

## Introduction

Clinical practice and clinical decision-making go hand in hand; it is virtually impossible to conduct work as a clinician and avoid making decisions that require practical application of clinical knowledge, problem-solving, weighing of probabilities, and balancing the risks and benefits of these decisions.^1^ Although decision-making is an inherent part of clinical practice, it is notoriously complex, as it consists of several, often complex and multi-target interventions, that need to be performed within the bounds of human capabilities as well as limits imposed by the modern health care system.^2^ Some of the specific challenges include (but are not limited to) the pressure to find the optimal solution, clinicians’ tiredness and uncertainty, proneness to heuristics and biases, information overload, inadequate staffing, and time constraints.^3,4^ Hence, it is not very surprising that a range of errors with varying severity can occur.^5^ During hospitalization, specifically, these most often include medication errors, such as dosing errors and medication omissions.^6,7^

Computerized clinical decision support systems (CDSSs) represent a paradigm shift in improving complex healthcare today.^8^ CDSSs refer to any electronic system designed to aid clinical decision-making, in which characteristics of patients are used to generate patient-specific assessments or recommendations that are then presented to clinicians for consideration.^9^ CDSSs are increasingly emphasized as one of the possible levers for improving care. For example, they have previously been linked to reduced medication errors, better accessibility and visibility of data, and faster prescribing of medication and treatment^10–13^. CDSS systems can be subdivided “knowledge-based” (i.e. rule-based) and “non-knowledge-based” (i.e. data-driven) with regards to how the medical knowledge is catured.^8,14^ Knowledge-based systems are workflow-driven and depend on expert rules that are used to process input data in order to produce an action or output. The rules are based on the extensive, subject-specific, clinical knowledge taken from the medical literature (i.e. literature-based), clinicians, and experts (i.e. practice-based), or patient-directed evidence.^15^ The non-knowledge-based systems are case-driven, however, they still require a data source. In fact, large sets of labeled data are required to train the algorithms.^16^ Namely, the “decisions” in the non-knowledge-based systems do not follow expert medical knowledge but leverage artificial intelligence (AI), machine learning, or statistical pattern recognition.^8,14^ The non-knowledge-based systems can simplify the knowledge acquisition and maintenance process.^17^ Such systems can even improve the accuracy and speed of diagnosis by analyzing complex patterns in high-dimensional data well beyond human performance^18–20^. However, in healthcare, where mistakes can cost human life, the non-explainable (i.e. black-box) nature of AI makes it unacceptable for clinicians and even regulators.^21^ In addition to doubts about systems, other barriers, such as lack of transportability and interoperability, their complexity and poor design (i.e. poor data quality and interpretation), privacy concerns and reluctance to use them in front of patients, poor computer skills and literacy, and deficient integration into existing workflows, prevent a wide adaption in practice.^8,10,22–24.^

As the benefits of CDSS can improve patient outcomes, it is vital to convert the barriers described above into facilitators of CDSSs’ use^25–27^. Hence, the present study protocol proposes the evaluation of a new system, developed with the aim to provide an information-rich, valid, safe, and easy-to-use tool that is well-integrated into the existing healthcare routine. The system will exploit patient-reported data, electronic health records (EHRs), and patient-reported outcomes (i.e. pain and psychological distress). The most prominent contribution of the proposed CDSS lies in its format of delivery and implementation. Specifically, the CDSS will be able to detect, as well as communicate, alarming situations (e.g. deviations in pain scores, heart rate, and blood pressure) during grand rounds and pre/post-operative care of patients with vascular and thoracic conditions. To this end, a fuzzy logic (FL)-based CDSS will be deployed. FL provides a transparent (explainable) and effective means for dealing with uncertainties in the health decision-making process.^28,29^ The rules will be defined based on expert knowledge and already established clinical guidelines and observations. Namely, the diseases were chosen as they are highly prevalent, have a typically complicated course of the disease, as there are multiple treatment options available and a lot of input information must be monitored.^30^ Furthermore, the newly developed CDSS will provide intuitive visualizations of patients’ health-related data, including EHRs, health quality measures (i.e. pain, blood pressure, oxygenation, and heart rate), patient-reported data, and patient-reported outcomes. The health data will be structured in an interoperable HL7-FHIR format.^31^ The real-time access during the grand round routine is ensured with speech-enabled user interfaces delivered via a tablet attached to the socially assistive humanoid robot (SAHR), Pepper.^32^ While the uptake of SAHR in clinical practice is so far limited,^33^ and to our best knowledge, there are no studies on the integration of CDSSs and social robotics yet, such innovative delivery could have several important benefits. In particular, it could make the use of a CDSS more effortless for clinicians (e.g. due to the robot being capable of movement, carrying the tablet, and offering assistance with queries in natural language) as well as more satisfactory and engaging for all involved stakeholders including patients.^34,35^ It could serve as an enabler of real-time querying and visualization of patient data.^36^ Adding human characteristics to the CDSS could contribute to higher trust in the system.^33,37,38^

We hypothesize that clinicians will accept the CDSS and recognize its clinical usability (H1). The use of CDSS will have a positive impact on health quality measures (H2) and the quality of life of patients (H3). In addition to our primary hypotheses, we aim to explore the impact of real-time access to the extended clinical background on diagnostic and treatment workflows for patients with vascular and thoracic diseases and conditions (R1).

## Methods

### Study design

The proposed study is designed as an experimental, prospective randomized controlled clinical trial. Participants of the study, that is patients with vascular or thoracic diseases, and clinicians, will be recruited at the regional university clinical center. The study will be carried out in one-week intervals over a two-year period, with one-week wash-out period between each iteration. Clinicians will be participating in the study over the whole two-year period, while patients will be participating only during their stay at the hospital. During wash-out period, the CDSS and SAHR are not used in the departments. The study will be carried out and reported on individually for the two cohorts. At the end of the study, the results will be cross-compared to assess possible generalizability across surgery settings.

Patients admitted to vascular or thoracic surgery wards for an elective (non-emergency) procedure will be screened for eligibility regarding the inclusion and exclusion criteria. Eligible participants will then be informed about the study characteristics and asked to fill out an informed consent form. Those who will consent to participate in the study will participate for five days (one week) and will be randomized by the clinicians into:

- Intervention Group (CDSS delivered by SAHR is actively used), and
- Control Group (CDSS is not used; instead, only standard workflow and progression charts are utilized).

**Figure 1.**
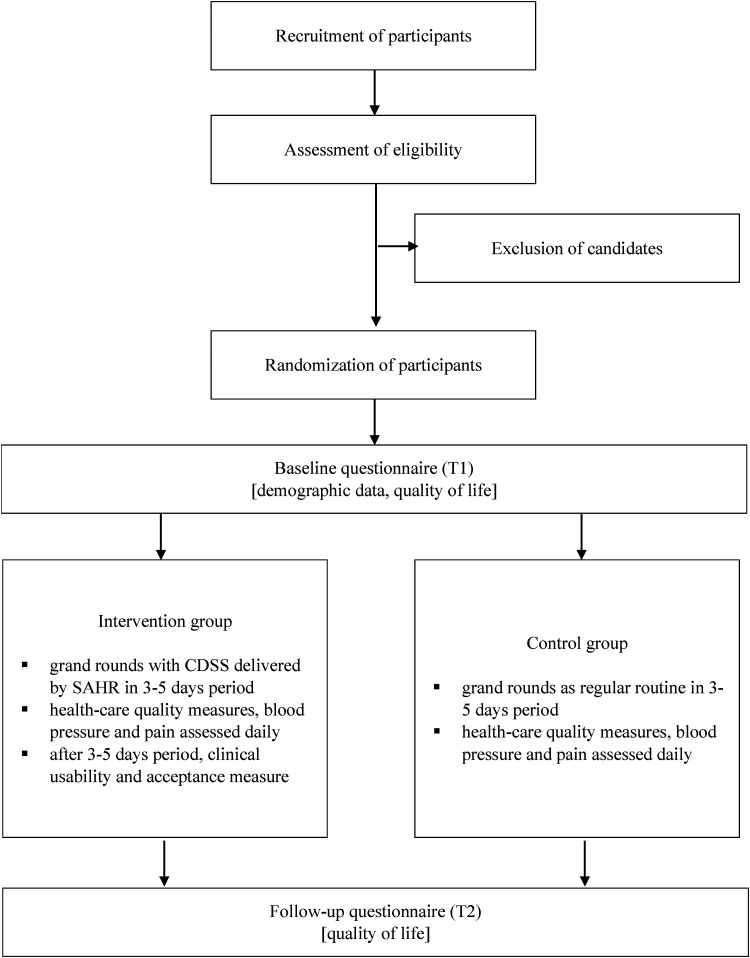
Study design.

Blinding will be achieved by handling the same tools during the grand rounds. However, in the control group, the robot and tablet will not provide any information to clinicians. Blinding will be furthermore achieved by adhering to the usual workflow and paper progression charts with all patients in both groups. The study procedure is outlined in Figure 1.

At the enrollment (T1), patients will fill out the baseline questionnaires collecting patient demographic information, and assessing their perceived quality of life. The perceived quality of life is again measured at the end of the intervention (T2).

Each patient will be followed for three–five days, depending on the standardized care. During this period, health care quality measures, including health quality measures, blood pressure, oxygenation and heart rate, and pain, are collected for both groups two times a day. Blood pressure, oxygenation, and heart rate are measured in all in-patients, recorded daily (standard procedure) and recorded on a progression chart. In the intervention group, blood pressure, oxygenation, and heart rate measures are stored electronically and the CDSS is used to flag deviations from the standard, and alert the treating clinician. In the control group, the standard progression chart is used as per hospital protocol. The treating clinician is autonomous to decide to step up or modify antihypertensive treatment. Pain is measured in all in-patients on daily basis, two times a day (standard procedure), and recorded on a progression chart. In the intervention group, the pain level is stored electronically and CDSS is used to flag and alert the treating clinician on pain levels identified above anticipated. In the control group, the standard progression chart is used as per hospital protocol. The treating clinician is autonomous to decide to step up or modify analgetic treatment.

The participating clinicians will be asked to evaluate the clinical usability and provide answers on their acceptance of the CDSS system five times during the study (after the first iteration, after half a year, after the first year, after a year and a half, and after the last iteration).

### Participants and recruitment

The proposed study intends to recruit two distinct samples: patients and clinicians. The patient sample will be composed of patients admitted to vascular surgery or thoracic surgery wards for an elective (non-emergency) procedure. Patients will need to be aged 18 years or above and willing to participate in the study. Exclusion criteria include emergency patients, patients already enrolled in other studies, patients with dementia, patients with special needs or appointed guardians, and patients allocated to an intensive step-down unit and/or regimen. The sample of clinicians will be composed of clinicians participating in a grand round routine within the vascular surgery or the thoracic surgery ward. Again, only employees aged 18 years or above, who will have signed a consent form, will be eligible to participate in the study.

There will be a pool of 400 eligible participants (200 per ward) available for inclusion in this study. Vascular and Thoracic Surgery Wards at the University Clinical Center Maribor treat over 4000 individuals annually and it is estimated that roughly 1400 individuals meet the inclusion criteria. However, based on the fact that, in general, at most 30% of participants (or less) consent to the clinical study,^39^ we assume that the final number of subjects willing to participate will be much lower. Regarding sample size requirements for the present study, public registries of clinical trials were reviewed. ClinicalTrials.gov returned 12 studies using the keywords “Vascular Diseases,” “CDSS,” and “randomized clinical trial,” while ISRCTN and EU Clinical Trials Registers returned no studies under the same conditions. These studies primarily focused on Stroke Prevention, CVD Risk Factor Control, and Hypertension. These studies differed substantially in the number of participants enrolled, from 40 to 400,000 participants per study. Considering the tertiary settings, the studies involved between 40 and 400 participants, with an average of 80 patients. In this study, 100 patients treated for Vascular Diseases will be enrolled in the study. Only one study was found on ClinicalTrials.gov using keywords “Thoracic Diseases,” “CDSS,” and “randomized clinical trial” involving 47 participants, while ISRCTN and EU Clinical Trials Registers returned no studies under the same conditions. In this study, 100 patients treated for Thoracic Diseases will be enrolled. Additionally, we also performed calculations using the G*Power software. Patient-related analyses will predominantly be based on the ANOVA repeated measures statistical test with the within-between interaction (two groups, two measurements). As we hypothesize a small effect size (*f* *=* 0.10) and intend to use a conventional significance threshold and power (α = 0.05, 1-β = 0.80), the total sample size needed to detect the effects is 200 participants.

The clinicians will be included in the study with the within-subjects design, whereby they will fill out the questionnaires five times during the study period. As such, we performed the sample size calculation in the G*Power software, choosing the Repeated Measures ANOVA test as the statistical test, entering a medium effect size (*f* = 0.25), and choosing a conventional significance threshold and power (α = 0.05, 1-β = 0.80). Such calculations suggest that at least 21 clinicians must be recruited overall.

### Measures

Testing the primary hypotheses will require the measurement of several outcomes, namely clinical usability and acceptance of the CDSS system from the clinicians’ perspective. To evaluate the clinical usability of the CDSS system, a System Usability Scale (SUS)^40,41^ will be used. SUS is an often-used measure for assessing the subjective usability of systems and products. Several studies proved its psychometric quality in several languages. It consists of 10 items (e.g. “I thought the system was easy to use.”), which are answered on a five-point scale from one (strongly disagree) to five (strongly agree).^42^

To evaluate the acceptance of the CDSS system, a unified theory of acceptance and use of technology and its extension will be used (UTAUT^43^ and UTAUT2^44^). The UTAUT model proposes four constructs regarding technology acceptance, that is performance expectancy, effort expectancy, social influence, and facilitating conditions. The extended version of the model, UTAUT2, incorporates additional three constructs, that is hedonic motivation, price value, and habit.^44^ As there is no one specific questionnaire based on the UTAUT and UTAUT2 models, but rather they are developed for specific studies and technologies assessed, this approach will be followed also in the present study in the co-creation phase, where the questionnaire will be defined in detail.

The secondary objective of the study is to evaluate the possible impact of the CDSS on health quality measures and quality of life of patients. To assess health quality measures, blood pressure, oxygenation and heart rate will be recorded daily, two times per day for all patients. In addition, pain levels will be assessed as part of the health quality measures. The visual analog scale (VAS) is a validated, subjective measure for acute and chronic pain. Scores are recorded by making a handwritten mark on a 10-cm line that represents a continuum between “no pain” and “worst pain.”^45^ VAS was evaluated with no statistically significant difference observed between the paper and laptop computer platforms. Thus, VAS can be adapted to digital and paper formats.

Finally, to evaluate the potential impact of CDSS on quality of life the EQ-5D-3L instrument^46^ is used in both groups of patients. The EQ-5D-3L's descriptive system comprises the following five dimensions: mobility, self-care, usual activities, pain/discomfort, and anxiety/depression. The EQ-5D-3L tool essentially consists of two pages: the EQ-5D descriptive system and the EQ visual analog scale (EQ VAS).^47^ The Slovenian version was validated by Prevolnik-Rupel with a representative sample of 3000 adults.^48^

## Plan of analysis

### Preliminary analyses

We will use the IBM SPSS Statistics 26 program for statistical analysis. In the first step, we will clean the dataset and exclude participants with missing data on any of the measurement points and within questionnaires, with more than 20% of missing values. We will also perform basic psychometric analyses of the questionnaires, namely the factor or other appropriate analyses to evaluate the internal structure of the questionnaires and analysis of reliability as internal consistency (coefficient α) and calculate the scores in accordance with the scoring instructions.

In the next step, we will perform basic descriptive analyses (means and standard deviations) and check the assumptions of the chosen statistical tests (such as the normality of the distribution assumption). This step will be followed by inferential tests, whereby results with a *p*-value below .05 will be considered statistically significant.

### Analyses of patient-related outcomes

The impact of CDSS use on the first patient-related outcome, that is health quality measures (H2), will primarily be analyzed with growth curve analyses.

The impact of CDSS use on the second patient-related outcome, that is perceived quality of life (H3), will primarily be analyzed with repeated-measure analysis of variance (ANOVA), with time as the within-subjects factor (T1 and T2; see Figure 1). Significant results will be followed up with pairwise post-hoc tests using the correction to adjust for multiple testing. All results will be accompanied by effect sizes.

### Analyses of clinicians-related outcomes

Clinicians’ acceptance of the CDSS and its perceived clinical usability (H1) will be analyzed with two separate repeated-measure ANOVA, with time as the within-subjects factor. Significant results will be followed up with pairwise post-hoc tests using the correction to adjust for multiple testing. All results will be accompanied by effect sizes.

### Other analyses

The additional research question (R1) will be assessed qualitatively through clinicians’ notes and observations given after the patient is released from hospital care.

## Discussion

Despite the growing need and interest for digital computer-aided support and many machine learning algorithms for classification and inference leading to automated interpretation of clinical data, their large-scale uptake is progressing rather slowly.^30,49,50^ This is especially disconcerting since existing studies suggest CDSS can contribute to patient safety,^7,51,52^ to adherence to clinical guidelines,^53^ and in management of patients on research/treatment protocols.^54^ Moreover, CDSSs can contribute to the optimization of healthcare costs by decreasing inpatient length-of-stay and reducing test duplication,^55^ and can even serve as computerized “consultation” or filtering (i.e. diagnostic decision support systems).^21^ One of the plausible reasons for this gap is low user acceptance.^56^ In the past, clinicians were not involved in the design and were consequently hesitant to accept CDSSs leading to suboptimal implementation. Furthermore, most interventions appear to achieve small to moderate improvements with the risk to cause alert fatigue or physicians’ burnout.^49^ It remains an industry-wide challenge to provide credible evidence, therefore, hindering wider adoption.^27^

Our study is hence designed to provide credible evidence on the impact of clinician-centric CDSS design and SAHR in the context of healthcare. More specifically, this study will provide crucial data on the effects of having access to extended clinical background on demand and within intuitive user interfaces delivered by SAHR during the grand round routine. As such, it will provide some answers regarding the practical value of deploying the solutions from a clinical perspective and their potential for improving health quality and quality of life among patients. At the same time, it seeks to investigate whether socially aware digital systems can reduce inpatient length of stay, increase medication efficacy, and reduce medication errors. We will conduct a rigorous study and use validated questionnaires to measure the outcomes. This will ensure the generation of high-quality evidence, on a large enough sample of patients and clinicians. Relevant recommendations and guidelines will be adhered to throughout the process. Special attention will be directed toward ethical considerations.

The proposed study is not without limitations. First, even though all surgery patients conforming to the inclusion criteria will be invited to participate in the study, it is plausible that only patients with more favorable attitudes towards technology will agree to participate in the study. This might lead to a sample that is generally younger^57^ and possibly favoring specific medical conditions.^58^ Second, due to the specific characteristics of vascular and thoracic surgery patients (e.g. length of hospitalization), their exposure to the modified routine will be somewhat short. Hence, the study will primarily focus on initial impact, whereas the trajectories of what happens over a more extended period (i.e. continued use), which can differ from initial reactions,^59^ will have to be explored in future studies. Lastly, in the present study, the new knowledge-based digital system will be limited to a few specific digital services, physical locations, one hospital, and decision support for two categories of diseases/conditions. Although capable of visualizing any clinical data, the clinician-centeredness and the FL support modules will be designed in collaboration with and around the needs of experts in the treatment and care of patients with the two specific conditions. This makes it difficult to provide general and final conclusions on the effectiveness of CDSS delivered with SAHR in hospitals.
